# Melatonin enhances sensitivity to fluorouracil in oesophageal squamous cell carcinoma through inhibition of Erk and Akt pathway

**DOI:** 10.1038/cddis.2016.330

**Published:** 2016-10-27

**Authors:** Yun-Xin Lu, Dong-Liang Chen, De-Shen Wang, Le-Zong Chen, Hai-Yu Mo, Hui Sheng, Long Bai, Qi-Nian Wu, Hong-En Yu, Dan Xie, Jing-Ping Yun, Zhao-Lei Zeng, Feng Wang, Huai-Qiang Ju, Rui-Hua Xu

**Affiliations:** 1 Sun Yat-sen University Cancer Center, State Key Laboratory of Oncology in South China, Collaborative Innovation Center for Cancer Medicine, Guangzhou 510060, China; 2 Department of Medical Oncology, Sun Yat-sen University Cancer Center, Guangzhou 510060, China; 3 Department of Pathology, Sun Yat-sen University Cancer Center, Guangzhou 510060, China

## Abstract

Oesophageal squamous cell carcinoma (ESCC) is the sixth most common cause of cancer-associated death in the world and novel therapeutic alternatives are urgently warranted. In this study, we investigated the anti-tumour activity and underlying mechanisms of melatonin, an indoleamine compound secreted by the pineal gland as well as naturally occurring plant products, in ESCC cells and revealed that melatonin inhibited proliferation, migration, invasion and induced mitochondria-dependent apoptosis of ESCC cells *in vitro* and suppressed tumour growth in the subcutaneous mice model *in vivo*. Furthermore, after treatment with melatonin, the expressions of pMEK, pErk, pGSK3β and pAkt were significantly suppressed. In contrast, treatment of the conventional chemotherapeutic drug fluorouracil (5-Fu) resulted in activation of Erk and Akt, which could be reversed by co-treatment with melatonin. Importantly, melatonin effectively enhanced cytotoxicity of 5-Fu to ESCC *in vitro* and *in vivo.* Together, these results suggested that inhibition of Erk and Akt pathway by melatonin have an important role in sensitization of ESCC cells to 5-Fu. Combined 5-Fu and melatonin treatment may be appreciated as a useful approach for ESCC therapy that warrants further investigation.

Oesophageal cancer ranks the eighth most-common cancer and is the sixth leading cause of cancer-associated death in the world.^1, 2, 3^ Oesophageal squamous cell carcinoma (ESCC), the major histological subtype comprises more than 80% of cases with oesophageal cancer^2, 3^ and occurs dominantly in Asia.^4^ Although advancements of early diagnosis intervention such as image-enhanced endoscopy and therapeutics such as endoscopic resection, surgery and chemoradiotherapy have led to improvements in clinical outcomes, only 15–25% of patients with ESCC could survive for 5 years after diagnosis.^5^ A majority of cancer patients eventually relapse or develop chemoresistance despite initial response.^1^ Therefore, it is urgent to develop effective chemosensitization agents to enhance the clinical efficacy.

Melatonin is an indoleamine compound mainly secreted by the pineal gland into the circulation to regulate chronobiological rhythms controlled by light and dark conditions and functions as immune modulators and reactive oxygen species (ROS) scavengers in diverse physiological activities.^6^ Recent studies also suggested that melatonin play oncostatic roles with its anti-proliferation and pro-apoptosis abilities.^7^ On the other hand, melatonin potentiate cytotoxicity of tamoxifen^8^ and gemcitabine^7^ or sensitize cancer cells to radiation^9^ without undesirable side effects. However, effects of melatonin on ESCC and in particular, the precise mechanisms have not been previously investigated.

Fluorouracil (5-Fu) is recommended as a key chemotherapeutic agent for ESCC patients.^1, 3^ However, only 30% of patients benefits from this therapy,^3^ to which detrimental side effects and chemoresistance are the most attributable. It has been well established that light exposure at night evinced suppression of melatonin is partially responsible for breast cancer progression and tamoxifen resistance.^8^ Moreover, some oral supplements^10^ or natural phytochemicals including melatonin^7^ have been shown to be able to reinforce the anti-neoplastic effects of anti-cancer drugs. Compelling data arising from tumour samples or *in vitro* studies have emerged to link aberrantly activated receptor tyrosin kinases (RTKs) with chemoresistance to 5-Fu,^11, 12^ and inhibitors of NF-κB and Akt have demonstrated synergism with 5-Fu in ESCC.^11, 13^ Meanwhile, melatonin has been reported to inhibit MAPKs, Akt and NF-*κ*B pathways.^7, 14^ These evidences prompted us to hypothesize that melatonin may enhance sensitivity to 5-Fu in ESCC cells.

Paramount reports have investigated two main signal transducers, namely the protein kinase B or Akt and the extracellular regulated protein kinase or Erk in various physiological and pathological conditions. Consistently, a growing body of evidences have revealed Erk as the effector regulated by the frequently hyperactivated RTKs including HER-2, EGFRs and playing roles in several oncogenic events including infinite proliferation and resistance to apoptosis in high percentage of human cancers.^15^ Similarly, Akt, signalled by PI3K and GSK3*β*, promotes tumorigenesis through phosphorylation of various downstream target genes including mTOR, thereby regulating cell survival, angiogenesis and metastasis.^16^ Genetic abnormalities in Erk and Akt pathways are common in human cancer, thus making Erk and Akt as well as downstream effectors promising targets for therapeutic intervention.^16^ For example, RTK/MAPK/PI3K pathway was found to be notably dysregulated in ESCC by high-throughput sequencing.^17^ In addition, Erk also cooperates with Akt as a determinant of cell fate, or form cross-talk networks regulated by Hippo/YAP pathway.^18^ Given their vital roles in oncogenic signalling transduction, it was thus reasonable to speculate that multitasking inhibitors of both Erk and Akt pathway would be effective and promising in cancer therapeutics.

This study was designated to explore the anti-cancer activity of melatonin against ESCC and whether the underlying mechanisms were associated with Erk and Akt pathway. Moreover, combinational effects between 5-Fu and melatonin on ESCC were also explored *in vitro* and *in vivo*.

## Results

### Inhibition of proliferation, migration and invasion of ESCC cells by melatonin

**Figure 1:**
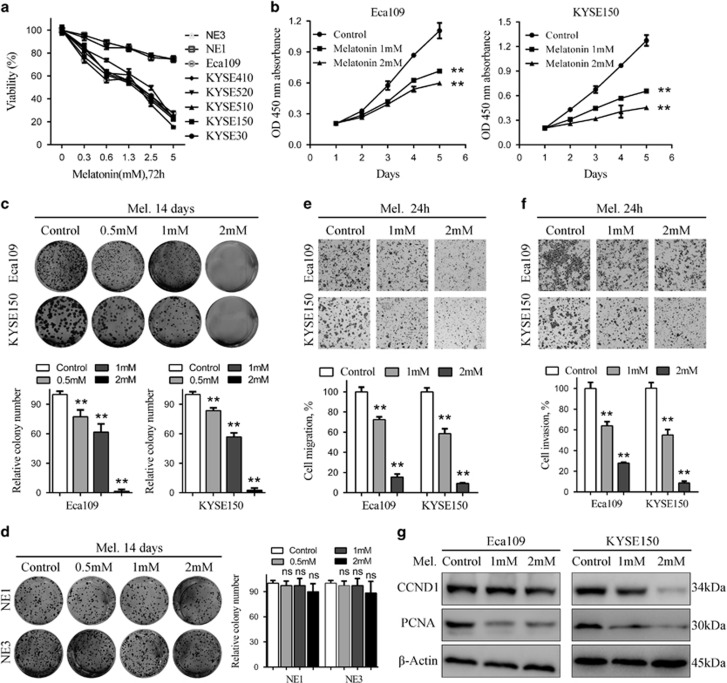
Melatonin inhibits proliferation, colony formation, migration and invasion of ESCC cells. (**a**) Viability of the indicated cells exposed to melatonin at different concentrations (72 h) was detected with MTS kit. (**b**) MTS assay of Eca109 (left panel) and KYSE150 (right panel) cells treated with melatonin (Control, 1 mM or 2 mM) at indicated time points. (**c**) Representative images (upper panel) and quantification (lower panel) of colony formation of the indicated cells cultured with melatonin at different concentrations for 14 days. (**d**) Representative images (left panel) and quantification (right panel) of colony formation of NE1 and NE3 cells cultured with melatonin at different concentrations for 14 days. Representative images and quantification of migration (**e**) and invasion (**f**) assay of the indicated cells treated with melatonin (Control, 1 mM or 2 mM) for 24 h. (**g**) Immunoblotting of CCND1, PCNA of cell extracts from Eca109 and KYSE150 cells after treated with indicated concentrations of melatonin for 24 h. *β*-Actin was used as loading control. Data in (**a**), (**b**), (**c**), (**d**), (**e**) and (**f**) are presented as mean±S.E. derived from three individual experiments with triplicate wells. **P<0.01 versus corresponding control. ns, no significant. Error bars, S.E.

To explore the effects of melatonin on ESCC cells, we used a panel of ESCC cell lines as well as two immortalized, non-cancerous NE1 and NE3 oesophageal epithelial cell lines. Melatonin, in a concentration range of 0–5 mM, decreased more than 70% of cell viability in ESCC cancer cells over 72 h continuous exposure as assessed by MTS assays, but only marginally reduced the viability of NE1 and NE3 cells (Figure 1a), indicating melatonin specifically inhibits viability of ESCC cancer cells. Moreover, melatonin at relative low concentrations suppressed cell viability of Eca109 and KYSE150 cells in a time-dependent manner (Figure 1b). To further explore the effects of melatonin on ESCC cells, the drug was removed after 72 h continuous treatment and the MTS assays indicated that melatonin consistently inhibited cell viability of Eca109 and KYSE150 cells 48 and 96 h after drug withdrawal (Supplementary Figure S1A). However, Eca109 and KYSE150 cells started to proliferate again at 96 h after drug withdrawal, indicating existence of melatonin-resistant cells and necessity of continuous melatonin treatment (Supplementary Figure S1A). Strikingly, colonies of both Eca109 and KYSE150 cells were nearly completely diminished after treatment with melatonin at 2 mM, while significant decrease was already observed at 0.5 mM (Figure 1c). Similar results were obtained in a panel of other ESCC cell lines including KYSE510, Eca18, KYSE30 and KYSE140 cells (Supplementary Figure S1B). However, melatonin exerted no inhibitory effects on colony formation of NE1 and NE3 cells, further demonstrating selective anti-tumour activity of melatonin in ESCC cells (Figure 1d). Transwell (Figures 1e and f) and wound healing assays (Supplementary Figure S1C) demonstrated that melatonin suppressed migration and invasion of Eca109 and KYSE150 cells. Meanwhile, treatment of melatonin at these concentrations (1 or 2 mM) for 24 h did not significantly reduce the viability of Eca109 and KYSE150 cells as shown in Figure 1b, which excluded the influence of melatonin-induced decreased viability to the results of cell migration and invasion. Further western blot analysis indicated that expressions of CCND1 and PCNA, proteins involved in proliferation, were inhibited after melatonin treatment in Eca109 and KYSE150 cells (Figure 1g). Collectively, melatonin selectively killed ESCC cancer cells and inhibited proliferation, migration and invasion *in vitro*.

### Induction of mitochondria-dependent apoptosis by melatonin in ESCC cells

**Figure 2:**
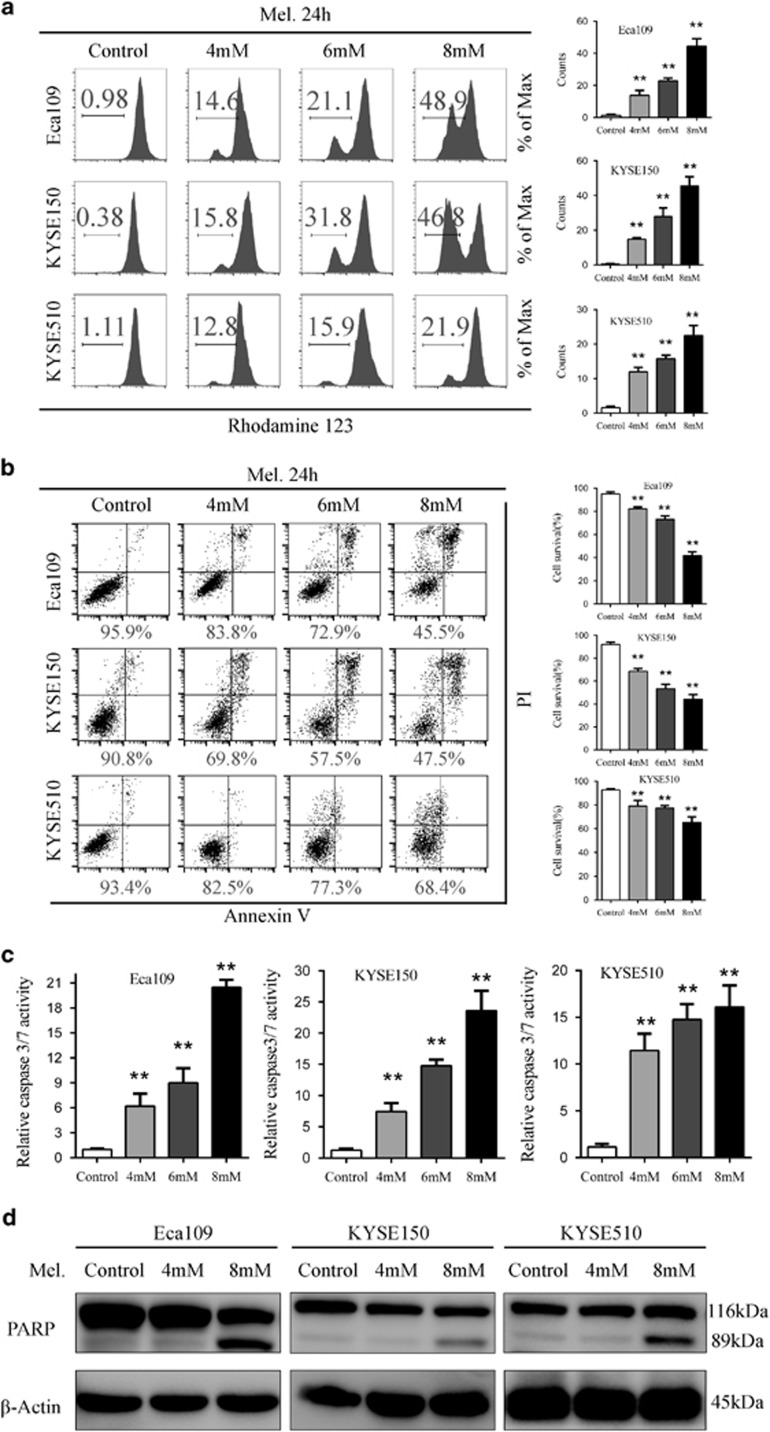
Melatonin induces mitochondria-dependent apoptosis of ESCC cells. (**a**) Representative images of mitochondrial transmembrane potential (left panel) and quantification (right panel) of cells negative for rhodamine staining in Eca109, KYSE150, KYSE510 cells treated with melatonin (Control, 4 mM, 6 mM) for 24 h. (**b**) Representative images of Annexin-V/PI assays (left panel) and quantification (right panel) of dual negative percentage in Eca109, KYSE150, KYSE510 cells treated with melatonin (Control, 4 mM, 6 mM, 8 mM) for 24 h. (**c**) Relative caspase 3/7 activity of Eca109, KYSE150 and KYSE510 cells treated with melatonin (Control, 4 mM, 6 mM, 8 mM) for 24 h. (**d**) Immunoblotting of PARP in the indicated cells treated with melatonin (Control, 4 mM, 8 mM) for 24 h. *β*-Actin was used as a loading control. Data in (a), (b) and (c) are presented as mean±S.E. derived from three individual experiments with triplicate wells. **P<0.01 versus corresponding control. Error bars, S.E.

Mitochondria-dependent apoptosis plays vital roles in the balance of cell proliferation and senescence.^19^ As apoptosis is often associated with collapse of mitochondrial transmembrane potential (Δ*Ψ*m), we analysed changes of Δ*Ψ*m by rhodamine staining and percentage of apoptotic cells with Annexin V and propidium iodide (PI) dual labelling after melatonin treatment via flow cytometry. With increase in the melatonin concentration, percentage of cells negative for rhodamine staining increased from 0.98, 0.38 and 1.11% in Eca109, KYSE150 and KYSE510 cells, respectively, to 48.9, 46.8 and 21.9% in those treated with melatonin at 8 mM (Figure 2a). Moreover, increased percentage of apoptotic cells paralleled with increased concentration of melatonin. Percentage of Annexin V/PI dual negative cells, namely survived cells after melatonin stimulus at 8 mM, was 45.5, 47.5 and 68.4% for Eca109, KYSE150 and KYSE510 cells, respectively (Figure 2b). To further confirm that melatonin induced mitochondria-dependent apoptosis in ESCC cells, we characterized effects of melatonin on activity of key apoptosis executioner including caspase 8 and caspase 3/7 and expression of cleaved PARP in ESCC cells. Activity of caspase 8 (Supplementary Figure S2) and caspase 3/7 (Figure 2c) was significantly increased after melatonin treatment. Furthermore, western blot analysis suggested a significant melatonin dose-dependent increase in cleaved PARP, the well-known characteristic of apoptosis (Figure 2d). To sum, melatonin induced mitochondria-dependent apoptosis of ESCC cells via caspase 8 and caspase 3/7 mediated PARP pathway.

### Melatonin suppresses activation of MEK/Erk and GSK3β/Akt pathway

**Figure 3:**
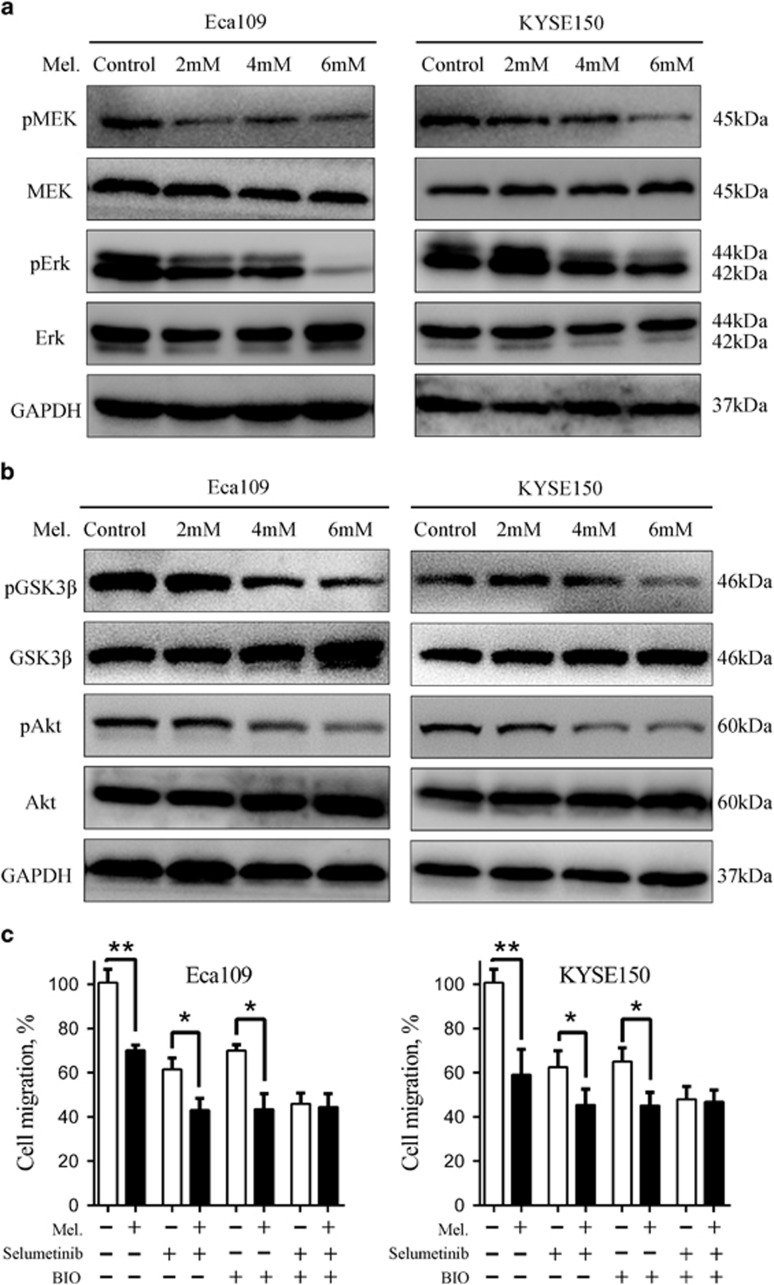
Melatonin inhibits MEK/Erk and GSK3β/Akt pathway in ESCC cells. (**a**) Immunoblotting of pMEK, MEK, pErk, Erk of cell extracts from Eca109 and KYSE150 cells after treated with indicated concentrations of melatonin for 24 h. GAPDH was used as a loading control. (**b**) Immunoblotting of pGSK3β, GSK3β, pAkt, Akt of cell extracts from Eca109 and KYSE150 cells after treated with indicated concentrations of melatonin for 24 h. GAPDH was used as a loading control. (**c**) Quantification of migration assays in Eca109 and KYSE150 cells treated with melatonin (1 mM), MEK inhibitor selumetinib (10 nM) or GSK3β inhibitor BIO (1 nM) for 24 h. Data in (**c**) are presented as mean±S.E. derived from three individual experiments with triplicate wells. *P<0.05 and **P<0.01 versus corresponding control. Error bars, S.E.

Because activation of Erk and Akt pathway has been implicated in tumorigenesis of various human cancers, we first evaluated expression of pErk and pAkt in a panel of ESCC cell lines and two immortalized oesophageal epithelial cells NE1 and NE3. Western blot analysis showed that pErk and pAkt was overexpressed in ESCC cell lines compared with that in NE1 and NE3 cells (Supplementary Figure S3A). Following immunohistochemistry assay of 15 paired ESCC cancer tissues and their matched adjacent non-tumorous tissues showed that although pErk was ubiquitously expressed in squamous epithelial cells (Supplementary Figure S3B), the tumour tissues exhibited a substantially stronger staining intensity (Supplementary Figure S3C). Our current observations combined with previous reports^11, 20^ prompted us to hypothesize that inhibition of Erk and Akt pathway may have therapeutic potential in ESCC treatment. We, therefore, tested whether activation of Erk and Akt was suppressed by melatonin in ESCC cells. As shown in Figure 3a, melatonin suppressed expression of pMEK, pErk, pGSK3β and pAkt in Eca109 and KYSE150 cells in a concentration-dependent manner. Moreover, exposure of Eca109 and KYSE150 cells to melatonin (5 mM) at different time points also decreased the expression of pErk and pAkt (Supplementary Figure S3D) and the expression of the upstream regulator pYAP (Supplementary Figure S3E). Additionally, pretreatment with MEK inhibitor (selumetinib) and GSK3β inhibitor (BIO) completely abrogated inhibitory role of melatonin on cell migration in Eca109 and KYSE150 cells, while either agent alone was not enough to attenuate anti-migration activity of melatonin (Figure 3c), further indicating that dual-inhibition of pErk and pAkt was involved in anti-neoplastic role of melatonin in ESCC. Altogether, melatonin suppressed activation of MEK/Erk and GSK3*β*/Akt pathway, which are aberrantly activated in ESCC.

ROS have been reported to be involved in anti-tumour activity of melatonin.^6^ To explore the effects of melatonin on the redox state of ESCC cells, we used the fluorescent probe 2',7'-dichlorofluorescein diacetate (DCF-DA) to monitor the intracellular ROS level in Eca109, KYSE150 and KYSE510 cells in the presence or absence of melatonin. As shown in Supplementary Figure S3F, melatonin induced ROS accumulation in ESCC cells and pretreatment with the anti-oxidant, N-acetyl-L-cysteine (NAC) significantly reduced the elevated ROS level. Moreover, pretreatment with NAC attenuated the pro-apoptosis effects of melatonin in Eca109, KYSE150 and KYSE510 cells (Supplementary Figure S3G). Taken together, induction of ROS production was also involved in the pro-apoptosis effects of melatonin in ESCC cells.

### Potentiation of cytotoxicity of 5-Fu by melatonin *in vitro*

**Figure 4:**
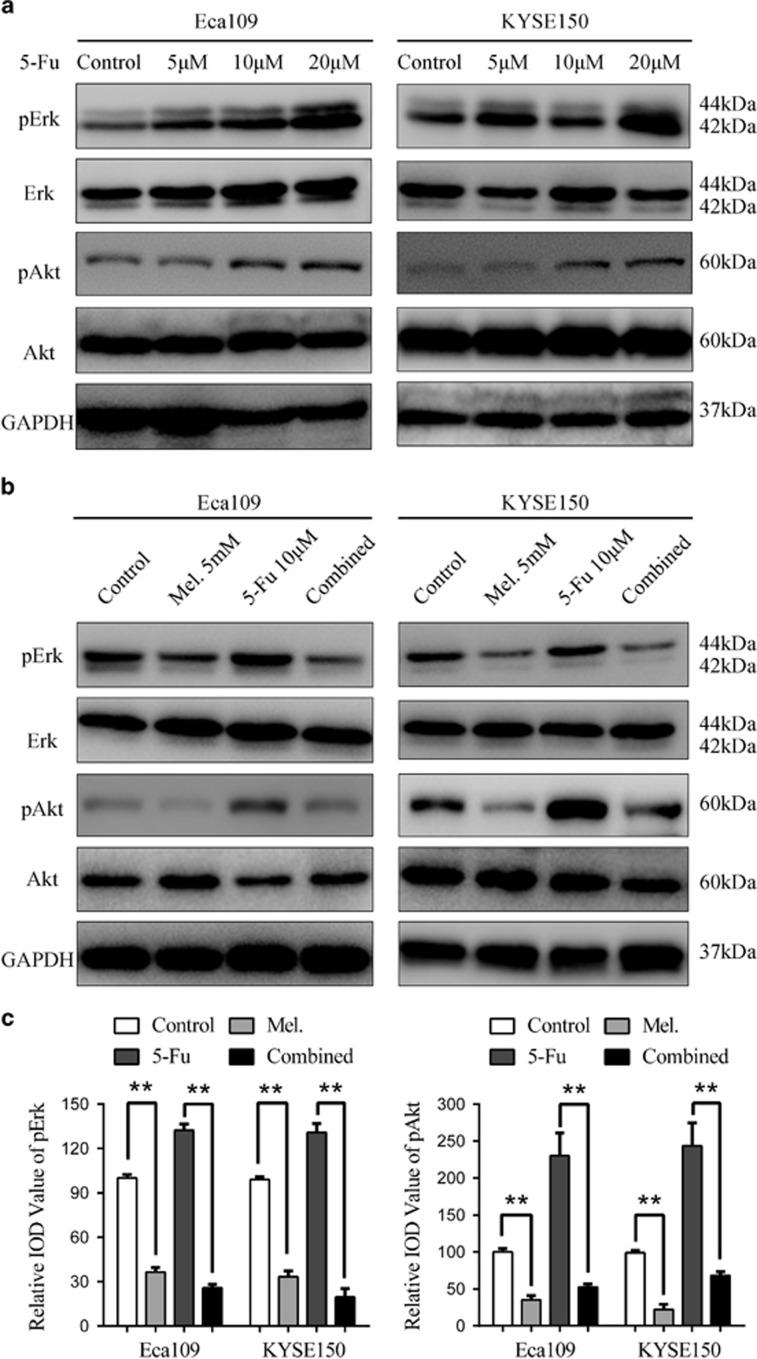
Melatonin inhibitis 5-Fu induced Erk and Akt phosphorylation. (**a**) Eca109 and KYSE150 cells were treated with 5-Fu (Control, 5 μM, 10 μM, 20 μM) for 24 h. Expression of pErk, Erk, pAkt, Akt was detected by western blot. GAPDH was used as a loading control. (**b**) Immunobloting of p-Erk, Erk, pAkt, Akt in Eca109 and KYSE150 cells treated with DMSO, melatonin (5 mM), 5-Fu (10 μM) or both agents for 24 h. GAPDH was used as a loading control. (**c**) Quantification analysis of pErk and pAkt expression in Eca109 and KYSE150 cells treated with DMSO, melatonin (5 mM), 5-Fu (10 μM) or both agents. Data in (**c**) are presented as mean±S.E. derived from three individual experiments with triplicate wells. **P<0.01 versus corresponding control. Error bars, S.E.

**Figure 5:**
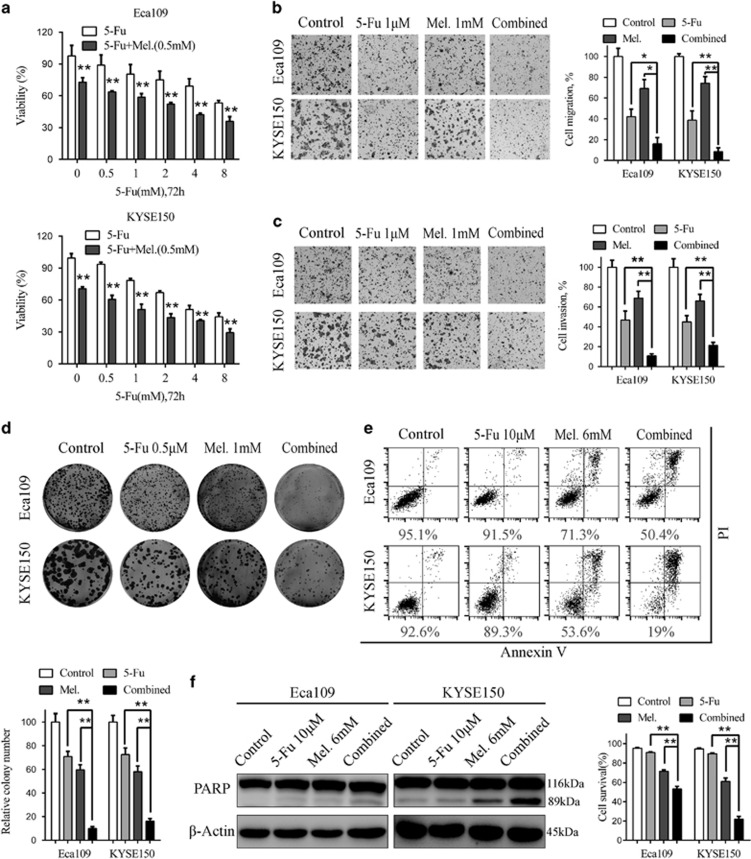
Synergistic effects between melatonin and 5-Fu in ESCC cells *in vitro*. (**a**) Cell viability of Eca109 (upper panel) and KYSE150 (lower panel) cells treated with 5-Fu alone or combined with melatonin (0.5 mM) at indicated concentrations was detected by MTS. (**b**) Representative images (left panel) and quantification (right panel) of migration assays in Eca109 and KYSE150 cells treated with 5-Fu (1 μM) and melatonin (1 mM) for 24 h. (**c**) Representative images (left panel) and quantification (right panel) of invasion assays in the indicated cells treated with 5-Fu (1 μM) and melatonin (1 mM) for 24 h. (**d**) Representative images (upper panel) and quantification (lower panel) of colony formation in Eca109 and KYSE150 cells treated with 5-Fu (0.5 μM) and melatonin (1 mM) for 14 days. (**e**) Representative images (upper panel) and quantification (lower panel) of Annexin-V/PI assays in the indicated cells treated with 5-Fu (10 μM) and melatonin (6 mM) for 24 h. (**f**) Immunoblotting of PARP in Eca109 and KYSE150 cells treated with 5-Fu (10 μM) and melatonin (6 mM) for 24 h. *β*-Actin was used as a loading control. Data in (**a**), (**b**), (**c**), (**d**) and (**e**) are presented as mean±S.E. derived from three individual experiments with triplicate wells. *P<0.05 and **P<0.01 versus corresponding control. Error bars, S.E.

5-Fu is one of the widely used chemotherapeutic drug for the treatment of several types of solid tumours including ESCC.^3^ However, detrimental side effects and chemoresistance have limited effects of 5-Fu.^3, 11^ Intriguingly, treatment of Eca109 and KYSE150 cells with 5-Fu resulted in elevated level of pErk and pAkt (Figure 4a), which could be reversed by combination of melatonin (Figures 4b and c). Melatonin also decreased the elevated pYAP expression induced by 5-Fu in ESCC cells (Supplementary Figure S4A). It was thus reasonable to speculate that melatonin could enhance sensitivity to 5-Fu in ESCC cells. First, MTS assays showed that combination of 5-Fu and melatonin significantly suppressed cell viability compared with 5-Fu alone (Figure 5a), demonstrating synergism as the combination index was less than 1 (Supplementary Figures S4B and C). Similar results were obtained in KYSE510 cells (Supplementary Figures S4D and E), while combination of melatonin and 5-Fu induced no synergistic effects in NE1 and NE3 cells (Supplementary Figures S4F and G). Second, cells migrated or invaded through the chamber significantly decreased after treatment with melatonin and 5-Fu compared with either agent alone in Eca109 and KYSE 150 cells (Figures 5b and c), which was further demonstrated with the wound healing assays (Supplementary Figures S5A and B). Furthermore, while 5-Fu at 0.5 μM impaired colongenic ability, addition of melatonin caused more dramatic decrease in colony numbers in Eca109 and KYSE150 cells (Figure 5d). Concordantly, significant fall of ΔΨm was observed following melatonin treatment as shown by rhodamine staining (Supplementary Figure S6A). Although apoptotic cells exposed to 5-Fu slightly increased compared to that exposed to dimethylsulfoxide (DMSO), cells survived in the combination group was significantly decreased compared to either agent alone (Figure 5e). Augmentation of 5-Fu induced apoptosis by melatonin was further confirmed in KYSE510 cells (Supplementary Figures S6B). In line with this, increment of caspase 3/7 activity and up-regulation of cleaved PARP were concurrently observed after treatment with melatonin and 5-Fu (Figure 5f, Supplementary Figures S6C). Altogether, melatonin enhanced sensitivity of ESCC cells to 5-Fu, possibly via inhibition of 5-Fu induced pErk and pAkt expression.

### Melatonin suppressed ESCC growth, alone or in combination with 5-Fu *in vivo*

**Figure 6:**
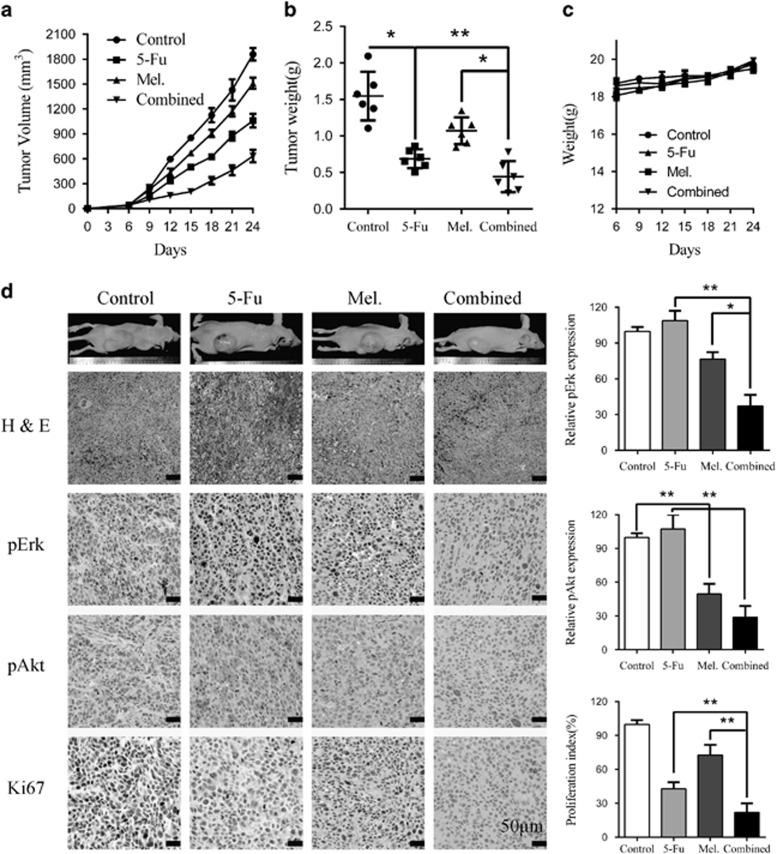
Melatonin enhance sensitivity to 5-Fu in ESCC cells *in vivo.* (**a**) Eca109 (1 × 10^6^/mouse) cells were subcutaneously inoculated into the dorsal flank of nude mice before they were treated with PBS, 5-Fu (20 mg/kg, twice per week), melatonin (20 mg/kg, once per day), or 5-Fu combined with melatonin. Tumour volumes were measured at indicated days. Data are shown as mean±S.E. of six mice in each group. (**b**) Excised tumour weight from the four separate groups was recorded. (**c**) Weight of the mice was recorded. (**d**) Left panel: representative hematein-eosin (H&E) and immunohistochemistry staining of pErk, pAkt, and Ki67 from tumour sections. Scale bar: 50 μm. Right panel: quantification of pErk, pAkt and Ki67 immunoreactivity in tumour sections. Data in (**a**), (**b**), (**c**) and (**d**) are presented as mean±S.E. (*n*=6). *P<0.05 and **P<0.01 versus corresponding control. Error bars, S.E.

To further investigate whether melatonin alone or in combination with 5-Fu suppresses ESCC tumour growth *in vivo*, we utilized subcutaneous xenograft mice model. Twenty-four BALB/c nude mice were inoculated with Eca109 cells (1 × 10^6^/mouse) in the dorsal flank. When the tumours were measurable 1 week later, the mice were assigned randomly into four groups that received melatonin, 5-Fu, combination of both agents or vehicle control for 4 weeks. The tumour volume was monitored twice per week. Melatonin significantly inhibited the growth of tumour xenografts (Figure 6a) with the tumour weight of melatonin-treated mice significantly less than that of negative control group (Figure 6b). Tumour growth as well as tumour weight in mice treated with melatonin plus 5-Fu was significantly suppressed compared with either agent alone (Figures 6a and b). By contrast, no weight loss or any other sign of toxicity was observed in any group (Figure 6c). The immunohistochemistry staining of excised tumour sections further revealed that melatonin suppressed elevated pErk and pAkt in the 5-Fu group (Figure 6d) consistent with the *in vitro* results. Ki-67 positive cells were significantly decreased in the combination group compared with single agent treated group (Figure 6d). To sum up, melatonin suppressed ESCC cell growth and overcame chemoresistance via inhibition of 5-Fu induced Erk and Akt phosphorylation *in vivo*.

## Discussion

The burden of ESCC has continuously increased amid the last decade.^1, 3^ Clinically, the majority of ESCC patients present with locally advanced disease, for which chemotherapy is widely used. However, undesirable side effects and acquired resistance have limited the effectiveness of chemotherapy or even result in disease recurrence. Herein, we investigated the putative potentiating effects of melatonin on chemotherapy-induced cytotoxicity in human ESCC cells *in vitro* and *in vivo*. In our present study, melatonin *per se* was able to display cytotoxic and pro-apoptotic activities towards ESCC via inhibition of Akt and Erk phosphorylation and enhanced sensitivity to 5-Fu in ESCC cells.

5-Fu, a pyrimidine analog, is widely used in cancer chemotherapy and displays anti-cancer activities through inhibition of thymidylate synthase, which is essential for *de novo* synthesis of thymidylate and subsequent incorporation into DNA.^21^ Neoadjuvant chemotherapy with 5-Fu plus cisplatin is widely recommended for ESCC patients, especially for those with locally advanced or distant metastatic lesions.^3, 22^ Moreover, preoperative chemotherapy with 5-Fu and cisplatin resulted in 12% increment of 5-year survival for stage II and III ESCC patients.^23^ However, intrinsic or acquired resistance to chemotherapy and undesirable detrimental effects have contributed to treatment failure and disease recurrence to some extent. Clinical and basic researchers have reported various mechanisms involved in 5-Fu resistance including dysregulated expression of genes participating in fluorouracil metabolism such as dihydropyrimidine dehydrogenase and thymidylate synthase,^24^ acquired mesenchymal transformation or stem-like characteristics,^25^ and activation of intracellular signalling pathways including EGFR/Akt.^12, 26^ We reported here that treatment of ESCC cells with 5-Fu leads to elevated phospholevel of Erk and Akt.

MAPK is a serine/threonine-specific protein kinase family responsible for various cellular activities including cell cycle, differentiation, cell survival and mitosis.^27^ Nonetheless, c-Jun N-terminal kinase (JNK) and p38, two members of MAPK family, are associated with apoptosis induction, while Erk plays a cytoprotective role^28^ and is linked to malignant transformation of human cancers. Phospholevel of Erk has been related to glial neoplasia,^29^ to progression and angiogenesis of melanomas^30^ and to breast cancer metastasis.^31^ In head and neck squamous carcinoma, levels of activated Erk correlated with higher nodal status and a higher proliferation rate and increased when tumour relapsed.^32^ With respect to ESCC, pErk was overexpressed in cancer cell lines and tumour tissues compared with their normal counterparts as detected by western blot and immunohistochemistry in our study, respectively (Supplementary Figures S3A and B), which is consistent with a recent report.^20^

Constitutively activated Akt pathway has been reported in many types of human cancers and associated with poor prognosis.^33, 34^ PI3K signals through phosphorylation of Akt, resulting in phosphorylation of adaptor proteins, transcription factors, cell cycle regulators and cancer susceptibility genes.^16^ As for ESCC, phosphorylated Akt was found to be overexpressed in tumour tissues compared with paired normal tissues^11^ and genetic variations of Akt predicts increased recurrence risk after chemoradiotherapy.^35^ Thus several inhibitors of PI3K/Akt pathway have entered preclinical as well as clinical trials.^16^ Exposure of breast cancer cells to PI3K inhibitors results in rapid suppression of MEK/Erk signalling, which is Ras-independent.^36^ On the other hand, a recent study has found that Akt-reactivation is MAPK/Erk2 dependent,^37^ indicating complex cross-talk and intersection of these pathways. Based on abovementioned reports and our findings that expression of pErk and pAkt was suppressed after melatonin treatment, its oncostatic activities against ESCC warrants further investigation.

Production of melatonin in the pineal gland is affected by light exposure and bottoms in the day and peaks at night.^6^ Mauricio F *et al.* recently identified seasonal fluctuations in day length-regulated melatonin production, which blocks Th17 differentiation and boosts Tr1 development, as immune regulator in multiple sclerosis.^38^ On the other hand, rise in breast cancer rates due to electrification of westernized lifestyle was postulated to be associated with disruption of night time melatonin production in the pineal gland.^39^ Anti-cancer activity of melatonin, alone or in combination with other therapeutic strategies, has been well established in several types of human tumours, such as breast^8, 40^ and pancreatic^7^ cancer. Specifically, involvement of Erk and Akt pathway has been documented with anti-inflammation and tumour-suppressive roles of melatonin.^8, 38, 40^ We found here that melatonin at micro molar level inhibits pErk and pAkt expression (Figures 3a and b), which was coincident with a previous report that melatonin inhibited breast cancer cell proliferation via suppression of Erk and Akt phosphoactivation.^40^ Phosphorylation-mediated inhibition of Erk and Akt was further found to be responsible for, at least in part, oncostatic activities of melatonin against ESCC as pretreatment with MEK and PI3K inhibitor completely attenuated anti-migration effects of melatonin on Eca109 and KYSE150 (Figure 3c).

ROS are byproducts of normal cell metabolism and reported to play vital roles in tumorigenesis and responses to anti-tumour therapy.^41^ The pro-oxidant or anti-oxidant roles of melatonin remain unclear.^42^ Melatonin exhibited potent anti-cancer activity against lung adenocarcinoma cells via induction of oxidative stress.^43^ However, treatment with melatonin effectively blocked glutathione depletion-induced apoptosis in Ras-transformed NIH3T3 cells via scavenging of free radical species.^44^ In our study, melatonin induced intracellular ROS accumulation in ESCC cells and pretreatment with NAC significantly attenuated the pro-apoptosis effects of melatonin, indicating that the redox modulation effects of melatonin is cell-type specific.

In conclusion, we explored the anti-proliferative and pro-apoptotic role of melatonin against ESCC in this study. Our results provided some evidence that melatonin suppressed phosphorylation of Erk and Akt, two significant pathways aberrantly activated in ESCC. Meanwhile, 5-Fu-induced activation of Erk and Akt could be reversed by co-treatment of melatonin, thus reinforcing its cytotoxicity against ESCC *in vitro* and *in vivo*. Therefore, preoperative treatment with melatonin, alone or together with 5-Fu, may be promising for clinical application in ESCC patients from the perspective of both efficiency and safety.

## Materials and Methods

### Cell lines and cell culture

Human ESCC cell lines KYSE520, KYSE410, KYSE150, KYSE30 were purchased from the Deutsche Sammlung von Mikroorganismen und Zellkulturen (DSMZ, Braunschweig, Germany). The ESCC cell lines Eca109, Eca18 and oesophageal epithelial cell NE1 and NE3 was a kind gift from Dc. Song LB from Sun Yat-sen University Cancer Center. The ESCC cell lines were grown in Dulbecco's modified Eagle medium (Invitrogen, Carlsbad, California, USA) supplemented with 10% fetal bovine serum (HyClone, Logan, Utah, USA) at 37 °C with 5% CO_2_. NE1 and NE3 cells were maintained in a 1:1 mixture of defined keratinocyte serum free medium with growth supplements and EpiLife medium with 60 μM Calcium (Invitrogen, Carlsbad, California, USA). All the cells were authenticated by short tandem repeat DNA fingerprinting and tested for mycoplasma before use at Medicine Lab of Forensic Medicine Department of Sun Yat-sen University (Guangzhou, China).

### Reagents and antibodies

Fluorouracil, selumetinib, BIO and NAC were purchased from Selleck Chemicals (Houston, TX, USA) and dissolved in DMSO or diluted water. DCF-DA was purchased from the Life Technology (Invitrogen, Carlsbad, California, USA) and dissolved in DMSO. Melatonin was from Sigma Aldrich (Sigma-Aldrich, St. Louis, USA). Antibodies include PARP, pAkt, Akt, pErk, Erk, GSK3*β*, pGSK3*β*, MEK, pMEK, *β*-Actin and GAPDH (Cell Signaling Technology, Beverly, MA, USA) as well as Ki-67 (Abcam, Cambridge, Massachusetts, USA).

### Tumour tissues microarray and immunohistochemistry

ESCC tissue microarray containing 15 paired cancer and para-tumour tissues was purchased from the OUTDO Bio Tech Co. (Shanghai, China). Sections were dewaxed by xylene before heated for 10 min at 95 °C in a microwave oven for antigen retrieval. Endogenous peroxidase activity was blocked by incubation with 0.3% H_2_O_2_ solution for 10 min. The sections were then incubated with antibodies against pErk (1:1000) at 4 °C overnight. Immunohistochemical staining was performed using a immunohistochemistry kit (Dako, Copenhagen, Denmark). No significant staining was observed in the negative controls, which were prepared by using the same class of immunoglobulin at the same concentration. To evaluate pErk protein expression, both the extent and intensity of immunoreactivity were assessed and scored, in which the scores of the extent of immunoreactivity ranged from 0 to 3 according to the percentage of cells that had positive staining in each microscopic field of view (0, <25%1, 25–50% 2, 50–75% 3, 75–100%) while the scores of intensity were as follows: 0, negative staining; 1, weak staining; 2, moderate staining; 3, strong staining. A total score was obtained by multiplying the scores for extent and intensity.

### Western blot analysis

Cells were treated with melatonin, 5-Fu or both before dissolved with radioimmunoprecipitation for protein extraction and separated by SDS-PAGE as previously described.^45^ Briefly, the cells were washed twice with cold PBS, scraped off the plate, pelleted and resuspended in radioimmunoprecipitation buffer. After lysis on ice for 15 min, samples were centrifuged and the supernatant collected. To quantify protein concentration of each sample, the BCA kit was used and equal amount samples were separated on 8–15% SDS-PAGE gels before transfferation to polyvinylidene fluoride membranes (Immobilon-P, Millipore, Bedford, USA). The membranes were then blocked with 5% non-fat milk in tris buffered saline tween (TBST) for 1 h at room temperature, incubated with the indicated primary antibody diluted in 5% bovine serum albumin in TBST at 4 °C overnight. Then the membranes were washed thrice with TBST, probed with peroxidase-linked secondary antibody for 1 h at room temperature. To visualize proteins in the membrane, enhanced chemiluminescence (SuperSignal ECL, ThermoFisher Scientific, Carlsbad, USA) was used.

### Cell proliferation and colony formation assays

Cell viability was measured with MTS (Qiagen, Hilden, German) assay according to manufacturer instructions. The absorbance was measured at wavelength of 490nm on a Synergy™ Multi-Mode Microplate Reader (Biotek, Vermont, USA). Colony-formation assay was carried out as described previously.^11^ Briefly, about 500 cells were seeded per well in six-well-plates 48 h before the addition of indicated chemicals. After 14 days, the cells were fixed in methanol and stained with 0.2% crystal violet. Number of colonies was counted using Quantity One software (Bio-Rad, Hercules, CA, USA).

### Transwell migration and invasion assays

Transwell chambers with or without matrix gel (Corning, New York, USA) were used to test effects of melatonin or 5-Fu on migration and invasion of ESCC cells as previously reported.^45^ Briefly, cells were pretreated with melatonin, 5-Fu or both, trypsinized and resuspended with medium without FBS. For the migration assay, 200 μl medium without FBS containing 2 × 10^5^ cells was added to the upper chamber, and 600 μl of medium with 50% FBS in the lower chamber acts as chemoattractant. For the invasion assay, 3 × 10^5^ cells suspended in 200 μl medium without FBS were added to the upper chamber with matrix gel, while 600 μl of medium with 50% FBS was added to the lower chamber. After culture for 24 h, chambers were fixed with methanol, stained by crystal violet (Sigma-Aldrich, St. Louis, USA) and the cells remaining in the upper chamber were removed with cotton swabs. Then the chambers were dried at room temperature and imaged using a microscope.

### Wound healing assay

Wound healing assays were used to detect cell migration ability. The cells were seeded in six-well plates before they were treated with the indicated chemicals. Then the 200- μl pipette tubes were used to create an artificial wound. The wound closure was photographed immediately and 24 h late under a microscope. We measured the fraction of cell coverage across the line for the migration rate.

### Cell apoptosis, rhodamine assays and detection of intracellular ROS

Cell apoptosis and decrease of mitochondrial transmembrane potential induced by melatonin or 5-Fu was determined by AnnexinV/PI (KeyGEN, Nanjing, China) and rhodamine (Beyotime, Shanghai, China) staining, respectively, followed by flow cytometer analysis (Beckman Coulter, California, USA) according to manufacturer's instructions. Also, caspase activity was measured by Caspase 3/7 Glo assay and Caspase 8 Glo assay (Promega, Madison, WI, USA) according to the manufacturer's protocol. The intracellular level of ROS was detected according to a previous report.^42^

### Animal study

To evaluate the anti-tumour effects of melatonin with or without 5-Fu, 24 female BABL/c nude mice (4–5 weeks old) from the Guangdong Province Laboratory Animal Center (Guangzhou, China) was used. Eca109 cells (1 × 10^6^) suspended in 100 μl cold PBS were subcutaneously injected to the dorsal flank of the mice. One week later, the mice were randomly assigned into the following different groups: Control, PBS; Melatonin, 25 mg/kg, once per day; 5-Fu, 20 mg/kg, twice per week; Combined, 5-Fu, 20 mg/kg, twice per week accompanied by melatonin, 25 mg/kg, once per day. Tumour volumes and mice weight were recorded twice weekly. After treatment for 4 weeks, the tumours were extracted from sacrificed mice, embedded in paraffin and sectioned. Our animal study was approved by the Institutional Animal Care and Use Committee of Sun Yat-Sen University. Tumour tissues from the BCLB/c nude mice were stained with H&E or immunohistochemically with pErk, pAkt, cleaved caspase 3 or Ki-67 according to previously reported protocols.^45^

### Statistical analysis

All data are presented as mean±S.E. To compare the statistical differences between two groups, Student's paired or unpaired *t*-test was used. As for comparisons among more than two groups, one-way ANOVA and Newman Keul's multiple comparison tests were used with the GraphPad Prism software (San Diego, CA, USA). A *P*-value less than 0.05 was considered significant. The Calcusyn Biosoft (Ferguson, MO, USA) was used to calculate combination index of melatonin with 5-Fu.

## Abbreviations

ESCC: esophageal squamous cell carcinoma
MEK: mitogen-activated protein kinase kinase
Erk: extracellular regulated protein kinase
GSK3*β*: glycogen synthase kinase 3β
Akt: protein kinase B
5-Fu: fluorouracil
ER: endoscopic resection
ROS: reactive oxygen species
RTKs: receptor tyrosin kinases
NF-κB: nuclear factor kappa B
MAPKs: mitogen-activated protein kinases
HER-2: erb-b2 receptor tyrosine kinase 2
EGFRs: epidermal growth factor receptors
PI3K: phosphatidylinositol 3 kinase
mTOR: mechanistic target of rapamycin
CCND1: cyclin D1
PCNA: proliferating cell nuclear antigen
YAP: yes associated protein
MTS: 3-(4,5-dimethylthiazol-2-yl)-5-(3-carboxymethoxyphenyl)-2-(4-sulfophenyl)-2H-tetra-zolium, inner salt
Δ*Ψ*m: mitochondrial transmembrane potential
PI: propidium iodide
PARP: poly (ADP-ribose) polymerase
DCF-DA: 2',7'-dichlorofluorescein diacetate
NAC: N-acetyl-L-cysteine
Ki-67: antigen identified by monoclonal antibody Ki-67
TBST: tris buffered saline tween
