# Supplementary material for: Melatonin enhances sensitivity to fluorouracil in oesophageal squamous cell carcinoma through inhibition of Erk and Akt pathway
Source: Cell Death Dis. 2016 Oct 27;7(10):e2432–. doi: 10.1038/cddis.2016.330 (PMC5133993; doi:10.1038/cddis.2016.330)
Supplement: Supplementary Figure Legends [file cddis2016330x1.docx]

**Figure S1:** **Melatonin suppressed cell viability, colony formation and migration in ESCC cells.** (A) Cell viability of Eca109 (upper panel) and KYSE150 (lower panel) cells after removal of melatonin in the medium. (B) Representative images (left panel) and quantification (right panel) of colony formation of KYSE510, Eca18, KYSE30 and KYSE140 cells cultured with melatonin at different concentrations for 14 days. (C) Representative images and quantification of wound closure in Eca109 and KYSE150 cells after treatment with melatonin. Data in A, B and C are presented as mean ± S.E. derived from three individual experiments with triplicate wells. *P < 0.05 and **P < 0.01 versus corresponding control. Error bars, S.E.

**Figure S2: Melatonin induced activation of caspase 8.** Relative caspase 8 activity of Eca109, KYSE150 and KYSE510 cells treated with melatonin (Control, 4mM, 6mM, 8mM) for 24h. Data are presented as mean ± S.E. derived from three individual experiments with triplicate wells. **P < 0.01 versus corresponding control. Error bars, S.E.

**Figure S3:** **Melatonin suppressed expression of pErk and pAkt and acts as pro-oxidant in ESCC cells.** (A) Western blot analysis showed that levels of pErk and pAkt in four ESCC cell lines (KYSE520, KYSE410, KYSE150 and Eca109) were higher than those in two esophageal squamous epithelial cells (NE1 and NE3). (B) Representative images of pErk immunohistochemistry in two paired cancer and normal tissues. Scale bars: 50μm. (C) Scores of pErk immunoreactivity in paired cancer and normal tissues. (D) Immunoblotting of pErk, Erk, pAkt, Akt of cell extracts from Eca109 and KYSE150 cells treated with melatonin (5mM) at different time points. GAPDH was used as a loading control. (E) Immunoblotting of of pYAP, YAP of cell extracts from Eca109 and KYSE150 cells treated with melatonin (5mM) at different time points. β-Actin was used as a loading control. (F) Representative images (left panel) and quantification (right panel) of relative DCF-DA level in Eca109, KYSE150 and KYSE510 cells exposed to melatonin at 2mM for 24h with or without pretreatment with NAC. (G) Apoptosis of Eca109, KYSE150 and KYSE510 cells exposed to melatonin (8mM, 24h) with or without pretreatment with NAC was detected by Annexin V/PI staining. Data in C are presented as minimum to maximum (n=15). *P* =0.03 tumor versus normal. Data in F and G are presented as mean ± S.E. derived from three individual experiments with triplicate wells. **P < 0.01 versus corresponding control. Error bars, S.E.

**Figure S4:** **Melatonin induced synergistic effects with 5-Fu.** (A) Immunoblotting of pYAP, YAP of cell extracts from Eca109 and KYSE150 cells treated with melatonin (5mM), 5-Fu (10μM) or both for 24h. GAPDH was used as a loading control. Fractional effect of melatonin treatment in Eca109 (B) and KYSE150 (C) cells. (D) Cell viability of KYSE510 cells treated with 5-Fu alone or combined with melatonin (0.5mM) at indicated concentrations was detected by MTS. (E) Fractional effect of melatonin treatment in KYSE510 cells. Cell viability of NE1 cells (F) and NE3 cells (G) treated with 5-Fu alone or combined with melatonin (0.5mM) at indicated concentrations was detected by MTS. Data in D, F and G are presented as mean ± S.E. derived from three individual experiments with triplicate wells. **P < 0.01 versus corresponding control. Error bars, S.E.

**Figure S5:** **Melatonin synergize with 5-Fu to inhibit migration of ESCC cells.** Representative images (left panel) and quantification (right panel) of wound closure in Eca109 cells (A) and KYSE150 cells (B) after treatment with melatonin (2mM), 5-Fu (1μM) or both. Data in A and B are presented as mean ± S.E. derived from three individual experiments with triplicate wells. *P < 0.05 and **P < 0.01 versus corresponding control. Error bars, S.E.

**Figure S6: Melatonin potentiates cytotoxicity of 5-Fu in ESCC cells.** (A) Representative images of mitochondrial transmembrane potential (left panel) and quantification (right panel) of cells negative for rhodamine staining of Eca109 and KYSE150 cells treated with melatonin (6mM) and 5-Fu (10μM) for 24h. (B) Representative images (left panel) and quantification (right panel) of apoptosis detected by Annexin V/PI staining after treatment with 5-Fu (10μM) alone or combined with melatonin (6mM) for 24h in KYSE510 cells. (C) Relative caspase3/7 activity of Eca109, KYSE150 and KYSE510 cells treated with 5-Fu(10μM) alone or combined with melatonin (6mM) for 24h. Data in A, B and C are presented as mean ± S.E. derived from three individual experiments with triplicate wells. *P < 0.05 and **P < 0.01 versus corresponding control. Error bars, S.E.
