# Supplementary figures and images for: Melatonin enhances sensitivity to fluorouracil in oesophageal squamous cell carcinoma through inhibition of Erk and Akt pathway
Source: Cell Death Dis. 2016 Oct 27;7(10):e2432–. doi: 10.1038/cddis.2016.330 (PMC5133993; doi:10.1038/cddis.2016.330)

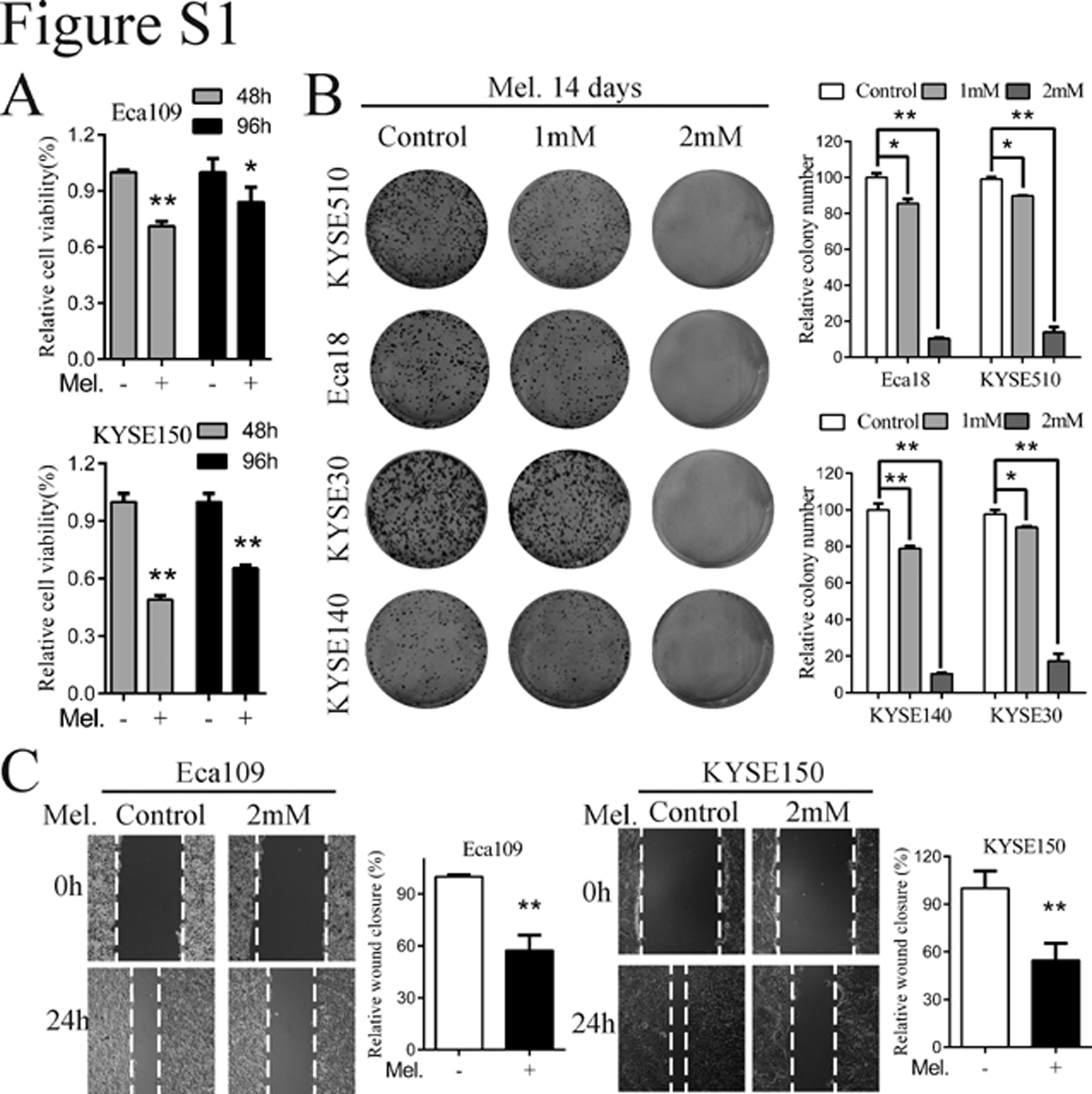

Supplement: Supplementary Figure 1 [file cddis2016330x2.tif]

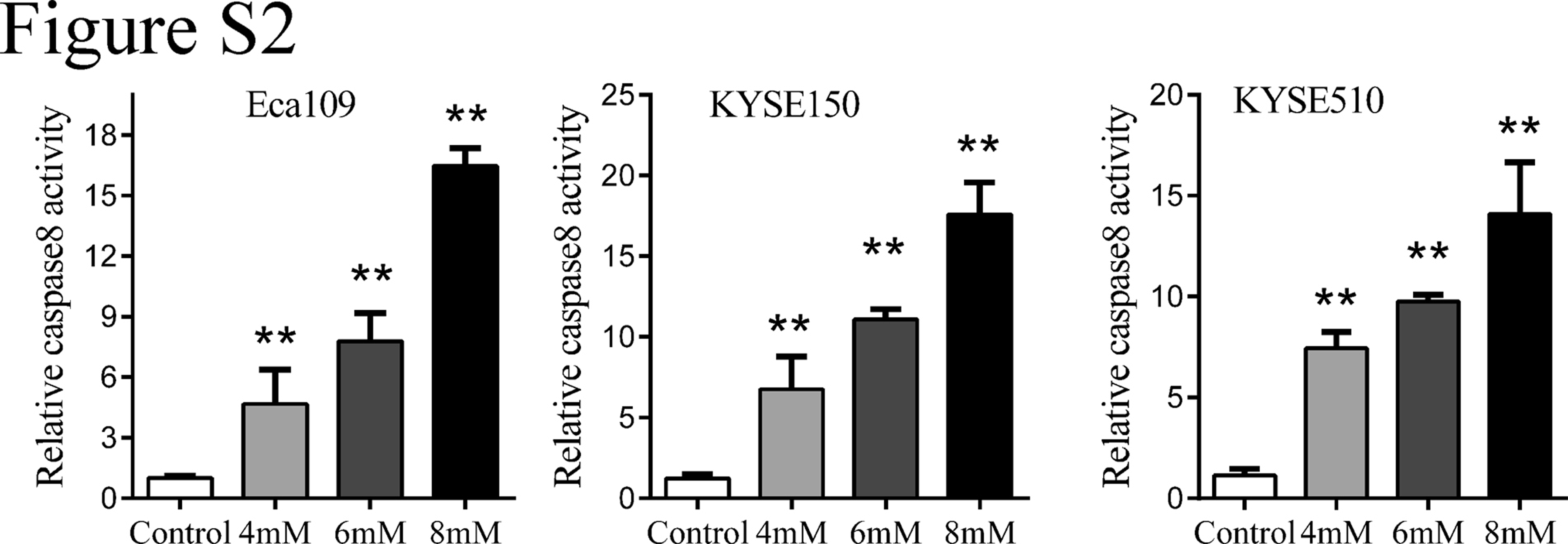

Supplement: Supplementary Figure 2 [file cddis2016330x3.tif]

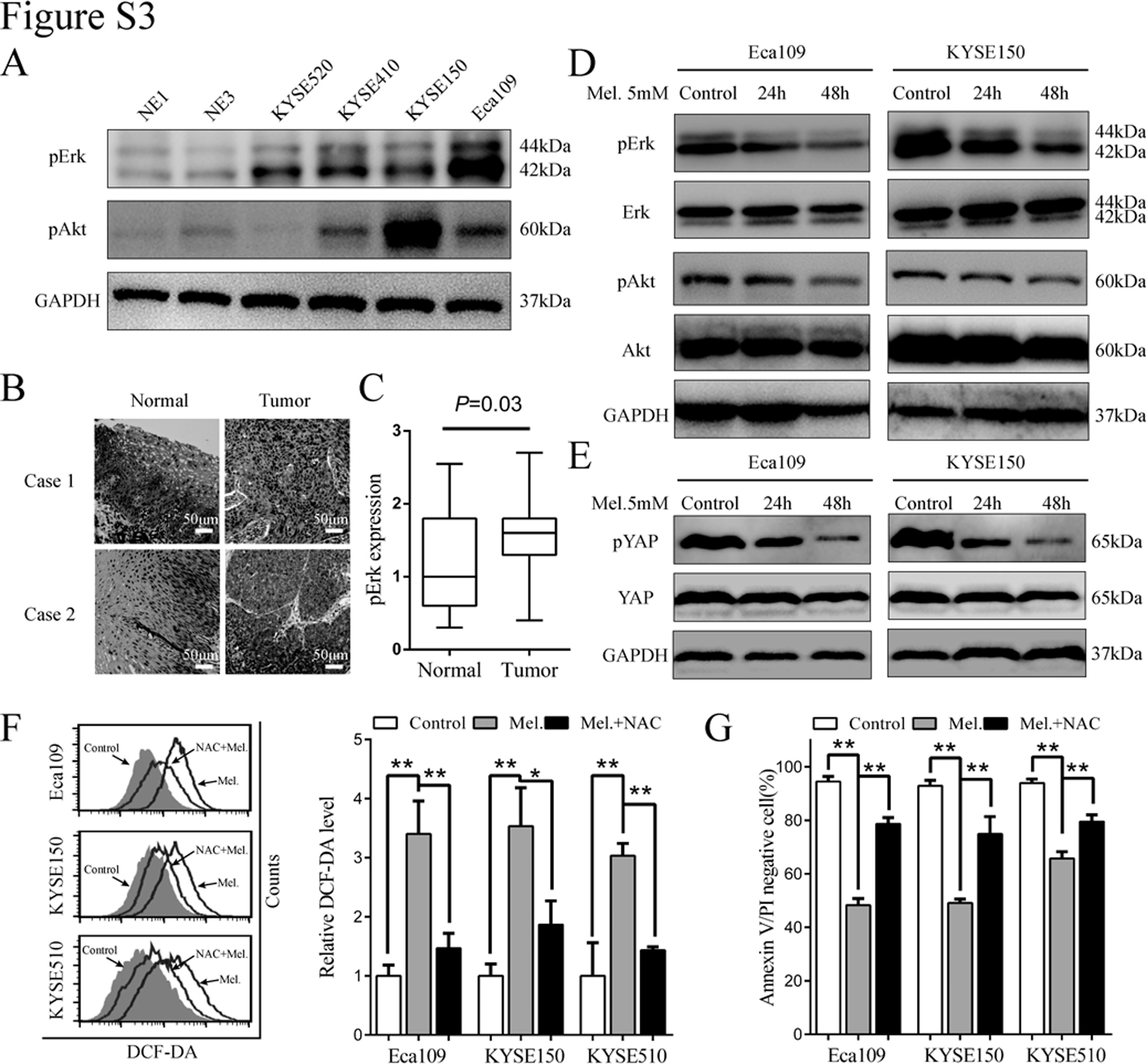

Supplement: Supplementary Figure 3 [file cddis2016330x4.tif]

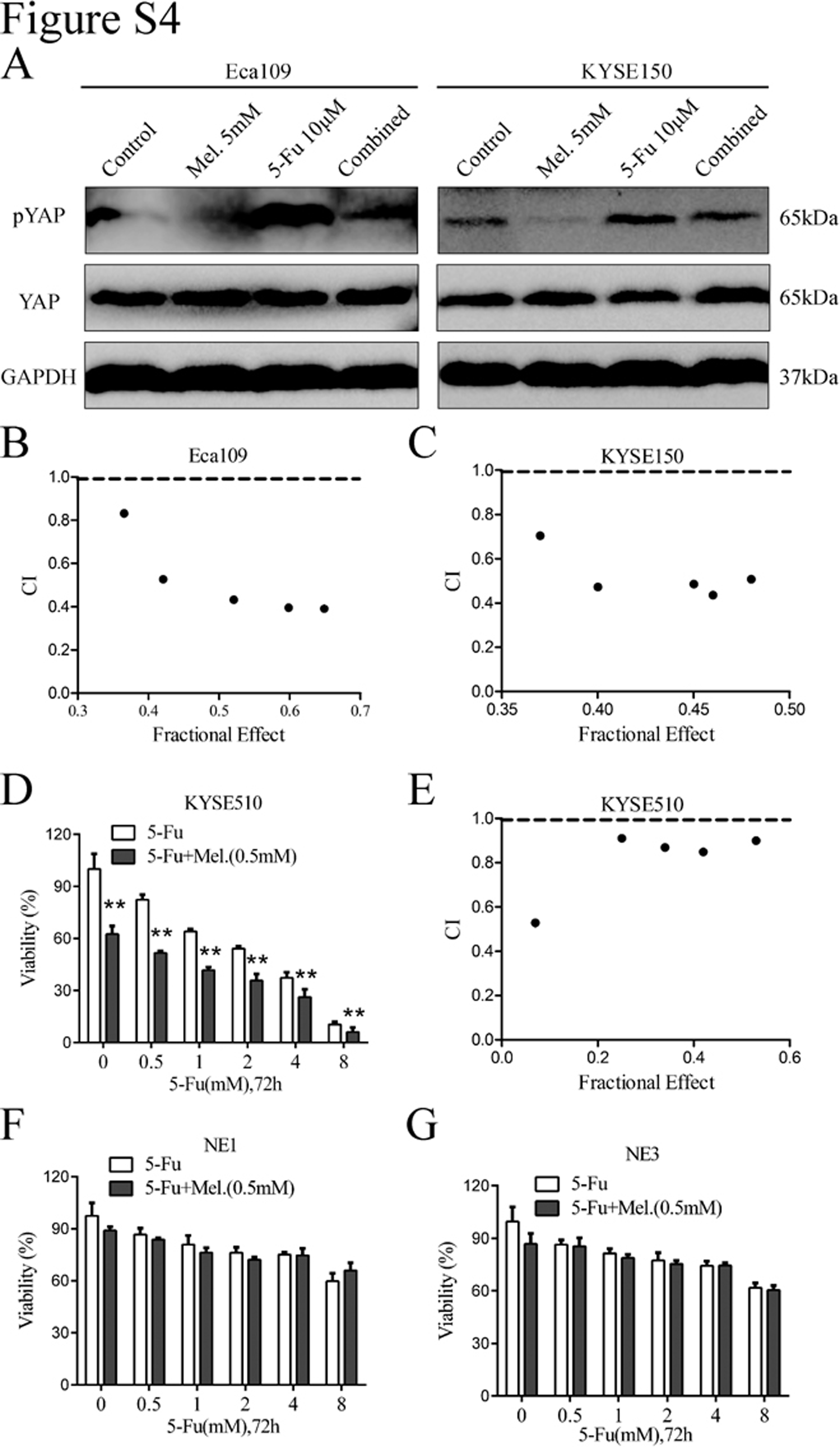

Supplement: Supplementary Figure 4 [file cddis2016330x5.tif]

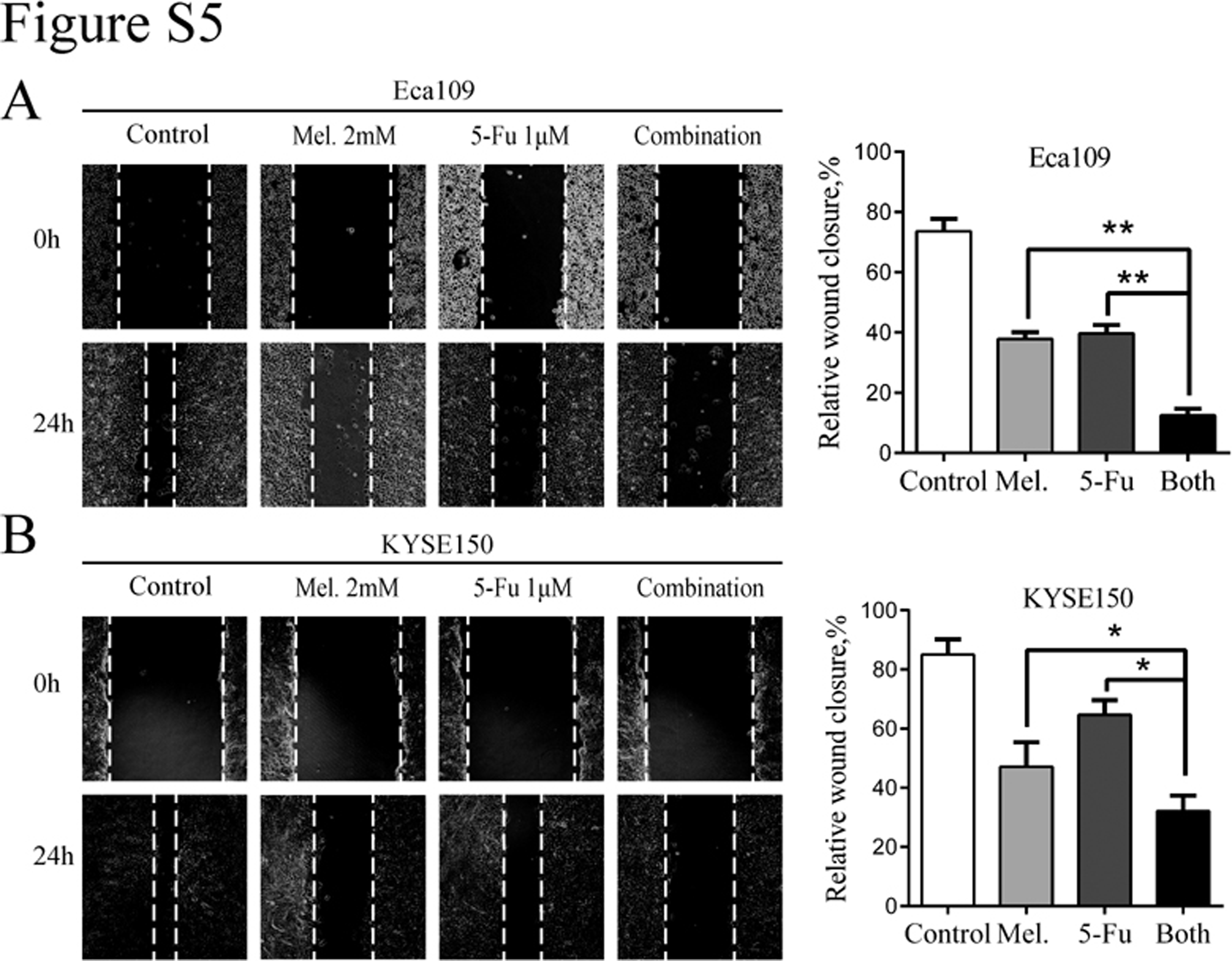

Supplement: Supplementary Figure 5 [file cddis2016330x6.tif]

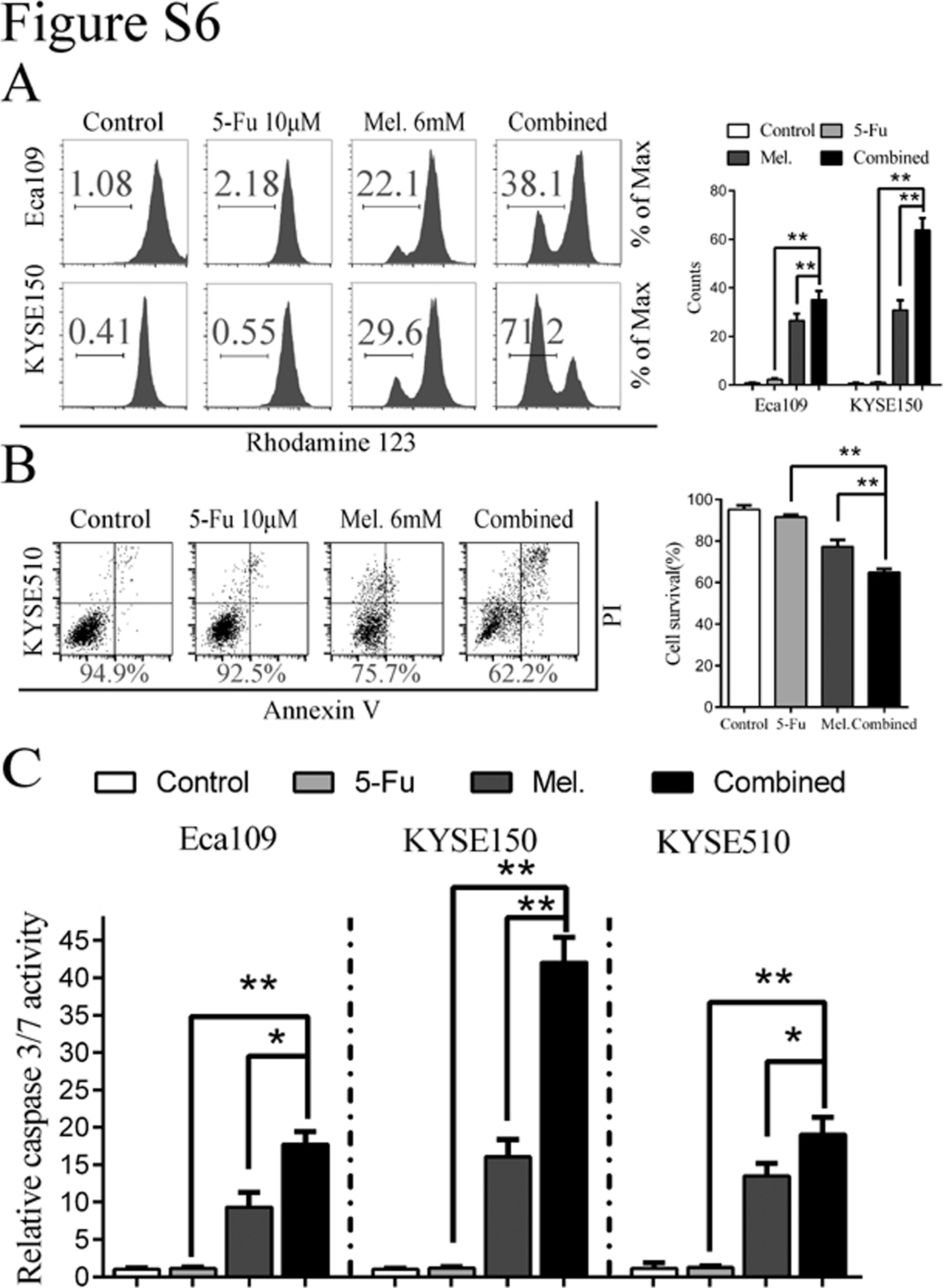

Supplement: Supplementary Figure 6 [file cddis2016330x7.tif]
